# Diabetes and associated cognitive disorders: Role of the Hypothalamic-Pituitary Adrenal axis

**DOI:** 10.1016/j.metop.2022.100202

**Published:** 2022-07-31

**Authors:** Nathalie Marissal-Arvy, Marie-Pierre Moisan

**Affiliations:** a INRAE, Laboratoire de Nutrition et Neurobiologie Intégrée, UMR 1286, UFR de Pharmacie, 146 Rue Léo Saignat, 33076, Bordeaux Cedex, France; b University of Bordeaux, Nutrition et Neurobiologie Intégrée, UMR 1286, 33000, Bordeaux, France

**Keywords:** Type 1 diabetes, Type 2 diabetes, HPA axis, Glucocorticoids, Memory, Hippocampus

## Abstract

Both diabetes types, types 1 and 2, are associated with cognitive impairments. Each period of life is concerned, and this is an increasing public health problem. Animal models have been developed to investigate the biological actors involved in such impairments. Many levels of the brain function (structure, volume, neurogenesis, neurotransmission, behavior) are involved. In this review, we detailed the part potentially played by the Hypothalamic-Pituitary Adrenal axis in these dysfunctions. Notably, regulating glucocorticoid levels, their receptors and their bioavailability appear to be relevant for future research studies, and treatment development.

## 1 Introduction

Diabetes is characterized by the inability of the body to produce or respond to insulin, with the consequence that the body cannot control the level of sugar in the blood, namely glycemia. The latest edition of the International Diabetes Federation (IDF) Diabetes Atlas shows that more than 460 million adults are currently living with diabetes, with increasing prevalence, which represents a strong socio-economic burden. There are two main types of diabetes. Type 1 Diabetes (T1D) is commonly diagnosed during childhood and adolescence and is characterized by the total lack of pancreatic cells producing insulin. T1D represents 5% of all diabetes types. Type 2 diabetes (T2D) is more common, often develops later in life, and is usually associated with metabolic syndrome. In T2D, high plasma glucose level is due to a default of insulin secretion from the pancreas, or a default in insulin action (insulin resistance). Diabetes show comorbidities such as cancer, infections, cardiovascular conditions or mental/cognitive disorders. Moreover, diabetes induces a background vulnerability that can facilitate the negative effects of these comorbidities [1]. This suggests an additive and/or synergistic relationship between risk factors of other diseases, consistent with the “diathesis” hypothesis of diabetes [2].

In the organism, the brain uses almost 20% of total plasma glucose. Glucose uptake in the brain is mainly insulin-dependent, and insulin receptors are widely distributed in the brain [3]. Brain function critically depends on glucose supply and insulin in the brain regulates several functions, such as whole-body energy metabolism in the hypothalamus, or memory formation in the hippocampus [4]. At childhood and adolescence, glucose requirement is increased for brain growth and development. Conversely, prolonged hyperglycemia induces neuronal damages in both humans and other mammals. In rodents, elevated glycemia imped myelin formation and neurotransmission [3], which can be critical according to the state of development. The term “diabetic encephalopathy” was introduced in 1950 as the expression of the central complications of diabetes [5,6].

Processes linking diabetes to cognitive dysfunctions have been recently investigated and the Hypothalamic-Pituitary Adrenal (HPA) axis appears as a key player in these processes. Here, we aimed to review cognitive impairments induced by diabetes, and the involvement of a high HPA axis in the cognitive dysfunctions induced by T1D and T2D.

## 2 Memory impairment in diabetes

### 2.1 T1D

Biessels et al. [7] suggest a ‘‘critical periods hypothesis” whereby neurocognitive deficits in T1D occur predominantly at two crucial periods of life, when the brain is developing in early childhood and when the brain undergoes neurodegenerative processes with ageing. Of note, the two positions are not mutually exclusive.

T1D occurs most often during childhood in humans when brain development is important. This implies that cognitive impairments following T1D can arise early in life [8]. Recently, Liu et al. showed that childhood-onset T1D was associated with an increased risk of neurodevelopmental disorders including attention-deficit/hyperactivity disorder, autism spectrum disorders and intellectual disability in a Swedish cohort [9]. Northam et al. [10] showed in children with T1D, deficits in general intelligence, speed of processing, learning capacity, attention, processing speed, long-term memory, and executive skills [11]. Cognitive dysfunctions are also observed in adolescents with T1D, impaired abilities of planning, adapting and reacting to environment, especially in concept formation, cognitive flexibility, anticipation, problem-solving capacity and word reading speed [[12], [13], [14]]. In these studies, T1D-induced cognitive alterations are independent of the quality of metabolic control and disease duration. In parallel, recent studies found associations between poor metabolic control and lower academic achievement, school performance, problem-solving capacity and cognitive flexibility [[15], [16], [17]], correlated with abnormal MRI data [18]. Aye et al. showed that a history of diabetic ketoacidosis was associated with changes in longitudinal cognitive and brain development [19]. Using a cross-sectional study design, cognitive and academic tests showed that young T1D subjects had lower verbal intelligence level compared to controls [17]. Increased exposure to hyperglycemia was associated to their lower spelling performance. Schoenle et al. [20] showed that impaired intellectual development in children with T1D was associated with glycated hemoglobin (HbA1c), age of diagnosis and sex.

Extreme hyperglycemic episodes, conducting to ketoacidosis (high levels of blood acids called ketones), affect strongly the brain [21], but chronic hyperglycemia has also been shown to be deleterious [20,[22], [23], [24], [25]]. Acute hyperglycemia negatively affected spatial working memory in adolescents [26], and chronic hyperglycemia is associated with smaller volume of grey matter in the right cuneus and precuneus, smaller white matter volume in the right posterior parietal region, and larger grey matter volume in the right prefrontal region [23,27]. In young children (4–10 years of age), alteration of grey matter related to hyperglycemia [28] have been showed in regions with rapid development, such as bilateral occipital and cerebellar regions, left inferior prefrontal cortex, insula and temporal pole region. For Cameron et al. [8] the cerebral consequences of a history of diabetic ketoacidosis and chronic hyperglycaemia appear to have been underestimated compared to hypoglycemia in children.

Adult patients suffering from T1D with poor metabolic control (Hba1c > 8.8%) show a psychomotor decline compared to those with better control (Hba1c < 7.4%) [6]. In a prospective study Ryan et al. [5] found that adults with T1D showed significant declines on measures of psychomotor efficiency compared to non-diabetic controls. Nevertheless, no difference was seen in the domains of learning, memory, or problem-solving tasks. At the same time, cross-sectional studies have shown that T1D subjects have performance deficits in multiple cognitive domains including information processing speed, psychomotor efficiency, memory, attention, academic achievement, visuospatial abilities and executive function [6,29]. Current and information processing abilities are poorer in young adults with early onset of diabetes (<7 years old) than those with later onset (7–17 years old) [30]. Nunley et al. [31,32] found in adult diabetic patients an incidence rate of cognitive impairments of 28% in comparison with 5% in controls. Even if cognitive deficits appear mild to moderate, they might hamper the day-to-day life, by limiting action of patients in more demanding situations [33]. Cognitive dysfunction appears inversely related to HbA1c, i.e. appropriate glucose control [34]. Diabetic brain features several symptoms best described as “accelerated brain ageing” as suggested by Biessels et al. [11]. Reduced white matter volume was associated with decreased cognitive performance by Wessels et al. [29], and microvascular complications such as retinopathy were also predictive of cognitive dysfunction. MRI measurements were not conclusive concerning a potential cerebral atrophy [33], nevertheless early diabetes onset has been associated with a higher ventricular volume [30,35].

The prolonged life span in T1D induces a risk for aging-related disease as dementia [32]. A retrospective study estimated that the risk ratio for dementia in T1D patients was 1.65 times compared to non-T1D subjects [32]. Neurodegenerative state interferes with daily life activities and increase the dependency of T1D patients to social assistance [32,36].

For McCrimmon, hypoglycemia and its rebound hyperglycaemia interact synergistically to improve oxidative stress and inflammation, damaging vulnerable brain regions and accelerating cognitive decline [37]. Poor management of hypoglycemia episodes is associated with smaller grey matter volume in the frontal lobe of children [27]. Kodl et al. [38] showed a correlation between reduced fractional anisotropy (representing neuronal connectivity), the duration of diabetes, and cognitive impairment in young adults. However, recurrent hypoglycemia, even severe, was not correlated with fractional anisotropy. Severe hypoglycemia was associated with smaller grey matter volume in the left superior temporal region [25]. T1D children experiencing severe hypoglycemia episodes before the age of five show deficits like delayed recall and spatial orientation skills [25,39]. Kaufman et al. [22] showed that stable glycemia improves cognitive abilities in young children. The hippocampus, a brain structure highly involved in spatial learning, is especially vulnerable to damage induced by hypoglycemia [27,[40], [41], [42]]. This provides a potential mechanism for learning and memory dysfunction occurring with T1D [43]. In T1D adults, when severe hypoglycemia occurs (seizures, even coma), studies have shown impaired attention [44], verbal skills [45], short-term visual-spatial and verbal memory [22,46], vigilance [47], full-scale or verbal intelligence scores [12].

In rodents, a model of T1D has been developed by treatment with streptozotocin (STZ). STZ is a glucosamine-nitrosourea which is taken up specifically by Glut-2 glucose transporter, highly expressed in pancreatic beta-cells [48] and absent in the blood brain barrier [49]. Injection of STZ induces a significant and rapid destruction of pancreatic beta cells. STZ-diabetic rodents are unable to produce insulin in sufficient quantities and suffer from exceedingly high glycemia (up to 600 mg/dl). STZ-diabetic rodents display polyuria, polydipsia and a strong delay in growth. In an ethical point of view, we do not advise researchers to keep STZ-T1D animals for a long time without insulin replacement [15,51]. For short diabetes duration (2–3 weeks), the measurement of plasma fructosamine concentration appears more relevant than HbA1c to characterize chronic hyperglycemia [51]. In STZ-T1D rat model, chronic hyperglycemia reduces neurons number and impedes myelination [52], and decreases spinogenesis and dendritic arborization inside the limbic system [53]. Both spatial learning and hippocampal long-term potentiation (LTP, reinforcement of synaptic contacts contributing to storage of information) are impaired, and enhancement of long-term depression (LTD, reduction of synaptic contacts causing the opposite effect of LTP) was measured in severely hyperglycemic rats. Both water-maze learning and hippocampal LTP are impaired in STZ-diabetic rats [54]. Repetitive hypoglycemia in young rat also impairs hippocampal LTP [11]. Early treatment substitution with insulin reversed STZ effects on Morris water-maze and hippocampal LTP, but not late treatment (10 weeks) [54]. In rat, Malone et al. show that chronic hyperglycemia could be more damaging to the developing brain than intermittent hypoglycemia [52], whereas this observation is still debated in human. Nevertheless, hypoglycemia was shown by Suh et al. [40] to induce transient neurogenesis (2 weeks) and subsequent progenitor cell loss (4 weeks) in the rat hippocampus, via a sustained activation of glutamate receptors in dentate gyrus. Studies in diabetic rats have suggested that ketoacidosis-induced chronic neuroinflammation could contribute to the cognitive decline of T1D individuals [55].

Recently, intracerebroventricular STZ injection (3 mg/kg) was shown to decrease cerebral glucose uptake (in particular in hypothalamus and circumventricular organs), and to produce multiple effects that mimic molecular, pathological and behavioral features of Alzheimer's disease (AD) [56]. This inability to metabolize glucose, and by extension AD linked to diabetes, have been called Type 3 Diabetes (T3D) [57]. STZ administration in rat lateral ventricles decreases brain glucose use in the frontal and parietal cortex, and by reducing ATP and phosphocreatine availability, diminishes energy charge potential in the cerebral cortex [21].

There is a strong association between T1D and cognitive impairments. It becomes more and more relevant to define the risk factors involved in this complication, and to search therapeutic targets on which we could act.

### 2.2 T2D

T2D is one of the most common chronic metabolic diseases. As a result of high calorie diets and sedentary lifestyles, diabetes is rapidly becoming more prevalent in Western societies. While global prevalence of diabetes in urban areas is 10.8%, in rural areas it is lower, at 7.2%. However, this gap is closing, with rural prevalence on the rise (IDF Diabetes atlas, 9th edition 2019). In addition to its well–known adverse effects on the cardiovascular and peripheral nervous systems, T2D also appears to negatively impact the brain, increasing the risk of depression and dementia [59]. Prospective studies showed that T2D people perform less in verbal memory, memory, information-processing speed, attention and executive function [[60], [61], [62]], in association with an hippocampal atrophy [27]. Mental flexibility and global cognition were not affected in all studies [63]. Cognitive decrements were associated with early onset of diabetes and poor glycemic control (ACCORD Memory in Diabetes study, MIND) [64]. Both genetic and environmental factors such as a lack of exercise, obesity, smoking, stress, and aging affect the development of insulin resistance which is involved in neurodegeneration [65,66]. MRI analyses identified structural atrophy (white, total grey matters, and volume of hippocampus [67], cortex or amygdala [68,69]), associated with neuropsychological deficits and vulnerability to dementia [70,71].

Experimental animal models of T2D show impairments in hippocampal-based memory performance [72], deficits in hippocampal neuroplasticity including decreases in dendritic spine density and neurogenesis [59] and decreases in synaptic transmission (LTP) [73], whereas bolstering insulin signaling mitigates β-amyloid-induced synapse loss in mature cultures of hippocampal neurons [72]. In a model of hippocampal-specific insulin resistance, rats showed deficits in LTP and spatial memory, especially long-term memory [74]. Central insulin administration improves spatial memory in a dose-dependent fashion in male rats [75], whereas intrahippocampal insulin microinjections enhances memory consolidation and retrieval [76]. Acute delivery of insulin into the rat hippocampus also promotes spatial memory in the alternation test [77], and transiently enhances hippocampal-dependent memory in the inhibitory avoidance test [78].

Aging is usually associated to insulin resistance, T2D and cognitive decline. Moreover, T2D was found associated with 50% increased risk of dementia [79,80], AD and vascular disorders [81]. Diabetes is now considered to be the second leading risk factor for AD, following aging itself [82]. Interestingly, Smolina et al. [36] measured an increased ratio for developing dementia in both diabetes (T1D and T2D) in a large cohort of patients in England.

T2D is often associated to weight excess. Proinflammatory cytokines produced by adipocytes can cross the blood-brain barrier and induce neuroinflammation, which favors subsequent neurodegeneration. Dietary manipulations appeared to ameliorate T2D alterations in periphery that were shown to be epidemiologically linked to a decreased incidence of AD and to retard pathogenesis in animal models of T2D [82]. Increased inflammation induces accelerated Aβ deposition [83] and/or decreased clearance and facilitates the polymerization of Tau. A recent study suggests that Tau could be preferentially involved in synaptic and cognitive deficits in T1D than in T2D experimental models [84]. Insulin signaling disorders also promote neuro-inflammation (notably elevated levels of cytokines/chemokines and gliosis [85]), apoptosis, oxidative stress, impairments of energy metabolism and synaptic disconnections [86], all of which lead to the development of cognitive impairment and AD, prompting some investigators to refer AD as T3D, characterized by an insulin-resistant brain state [87]. The concept of brain insulin resistance was advanced by Arnold et al. [88]. Interestingly, the brain regions with the highest densities of insulin receptors, such as the hippocampus and temporal lobe, are also the major targets of neurodegeneration in AD. Studies showed a strong genetic association between T2D and AD, with candidate genes involved in insulin resistance, and others leading to uncontrolled inflammatory stress on neuronal tissues, which can precipitate the formation of amyloid and Tau proteins, thus worsening AD development [83,89]. Recently, Kshirsagar et al. reviewed the role played by insulin resistance as the connecting link between AD and T2D [90].

To conclude, drugs that increase insulin sensitivity might have a positive effect on the cognitive consequences of diabetes. In this regard, members of the incretin family (notably Glucagon-like peptide-1 (GLP-1), exendin-4 or liraglutide) could be considered as prime candidates to delay cognitive decline, and even to improve the mild cognitive decline and AD symptoms [4,72]. Recently, brain derived neurotrophic factor (BDNF), dipeptidyl peptidase-4 (DPP4), and Ca2+ brain dysregulation were identified as novel biomarkers and potential therapeutic targets for diabetes-related cognitive impairment [91,92].

An intranasal treatment with insulin could be a promising perspective to prevent cognitive deficiencies induced by diabetes regardless of patient age. Insulin via this route of administration travels in convective bulk flow along perivascular pathways following the olfactory and trigeminal nerves and importantly by passing the blood brain barrier. In this way, insulin can reach the hippocampus and the cortex in 15–30 min [93,94]. Importantly, intranasal insulin does not reach the general circulation [95], thereby avoiding peripheral hypoglycemia. Intranasal insulin administration improves working memory in both human and animal studies, and intrahippocampal delivery of insulin improves hippocampal-dependent spatial working memory. This positive effect was due to regional vasoreactivity, especially vasodilatation in the anterior brain regions, such as insular cortex that regulates attention-related task performance [4]. Moreover, by inhibiting apoptosis, insulin promotes neuronal survival [88]. Novak et al. [96] demonstrated that intranasal insulin increases resting-state connectivity between the hippocampus and the medial frontal cortex compared to placebo.

## 3 Involvement of HPA axis in memory impairments induced by diabetes

Glucocorticoids (cortisol in humans and most mammals, corticosterone in rats and mice) are produced by the adrenal cortex and regulated by ACTH under the control of the HPA axis [97]. The major determinants of corticosteroid action are the level of free cortisol in the plasma and the densities of its receptors in target tissues. As little as 5% of cortisol circulates free in the plasma, with the majority bound with high affinity to corticosteroid binding globulin, which acts as a reservoir and a transporter of steroid to target cells, as well as lower affinity proteins such as albumin. The production of glucocorticoids is contingent upon the pronounced circadian rhythm: low during quiescence/sleep (2–10 ng/ml in male rat; 5–20 ng/ml in human), and high during the active phase (100–250 ng/ml in male rat; 50–150 ng/ml in human). Episodic stressful events stimulate HPA axis which can strongly increase glucocorticoids in blood (up to 200–300 ng/ml in male rat, and up to 500 ng/ml in human), and considerable variations in free plasma cortisol concentrations occur. Tissue glucocorticoid action is also determined by an intracellular enzyme that metabolizes glucocorticoids, the 11β-hydroxysteroid dehydrogenase type 1 (11β-HSD1, [98]). Cortisol is believed to diffuse across cell membranes and then to bind to cytoplasmic glucocorticoid receptors (GR), which then translocate to the nucleus. GR are ligand-gated transcription factors that regulate a plethora of genes directly or through interactions with other transcription factors. In some tissues, especially those involved in memory, glucocorticoids also bind mineralocorticoid receptors (MR) with a higher affinity than GR.

Due to their pivotal role in endocrine systems, glucocorticoids have been associated to vulnerability to many diseases, and to pathology complications. Taking into account the involvement of the HPA axis in neurobiology and memory, impairments in HPA function may exacerbate the neurobiological complications of both T1D and T2D [99].

### 3.1 Glucocorticoid levels and cognitive functions

Both absence and excessive levels of glucocorticoids appear deleterious for cognitive functions. Removal of glucocorticoids by adrenalectomy or synthesis inhibition by metyrapone impairs memory consolidation in rats [100,101], i.e basal levels of glucocorticoids are needed for neuronal maintenance. Conversely, elevated cortisol levels have been associated with poor cognitive ability in humans subjected to psychosocial stress [102], during normal aging [103] and in AD [104]. In rats, high glucocorticoids induces morphological changes in hippocampus (notably in CA3), and cognitive deficits [105]. An acute stressful experience decreases the number of adult-generated neurons in the dentate gyrus in various species, and hippocampal volume [106]. Moreover, chronic exposure to stress-induced elevations in corticosterone suppresses adult neurogenesis, reduces LTP, and impairs learning on hippocampus-dependent tasks in rats, such as behavior in the Y-maze [[107], [108], [109]]. Diamond et al. [110] reported an inverted U-shape relationship between the level of circulating corticosteroids, and LTP in the hippocampus of rats. The induction of LTP in the hippocampus is blocked by the administration of corticosterone, and there is a negative correlation between the magnitude of LTP in the hippocampal CA1 and the level of circulating corticosteroids [106]. According to Roozendal et al., glucocorticoid effects on memory also depends on catecholamines, notably in the basolateral amygdala [111]. Related to diabetes, it has been shown that memory deficits induced by chronic restraint stress are related to an impaired insulin signaling in mice, that is rescued by intranasal insulin treatment [112].

#### 3.1.1 T1D

Humans with poorly controlled diabetes exhibit disturbances in their HPA axis function, resulting in increased basal activity, high nocturnal rise in plasma cortisol, impaired negative feedback [113], and greater responses to CRH stimulation [114]. Similarly, levels of adrenal glucocorticoids are elevated in rodents with experimental diabetes, which has been linked to memory impairments and underlying dendritic reorganization [115]. These alterations are reversed by a return to basal glucocorticoid concentrations [108], or prevented by inhibiting glucocorticoid synthesis [116]. Importantly, these alterations are also reversed with insulin treatment [117]. Because glucocorticoid excess has been causally linked to decreased hippocampal neurogenesis and cognitive deficits in STZ-induced diabetic adult rodents, it was important to evaluate glucocorticoid regulation in juvenile models of T1D, regarding the mean age of occurrence of T1D in human. Elevated plasma concentration of corticosterone was detected in untreated diabetic rats [118], as expected with such a chronic metabolic stress, but only at the nadir of secretion [51]. In response to a restraint stress, the peak of secretion of corticosterone was similar between experimental groups, contrary to the recovery that was delayed in untreated diabetic rats [51], suggesting an impaired feedback mechanism as described in humans. Although insulin treatment prevented the increased levels of corticosterone in basal conditions, there was no beneficial effect under stress conditions [50]. Within hippocampus, corticosterone concentration was also increased at the nadir in untreated diabetic rats, as well as 11β-HSD1 activity. The impaired negative feedback observed after stress may involve an altered neuronal environment that could be related to microstructural and neurogenesis data [51]. It has been suggested that chronic high glucocorticoid levels are responsible for the functional and morphological degeneration occurring in the hippocampus of T1D rats and mice [59]. Indeed, Stranahan et al. [119] reported that impaired LTP, deficits in cognitive performance and reduced neurogenesis were normalized in diabetic rodents after adrenalectomy and corticosterone replacement at physiological concentrations [120].

#### 3.1.2 T2D

High glucocorticoid secretion is associated with T2D, and promotes gluconeogenesis in the liver, suppresses peripheral glucose uptake, enhances lipolysis, decreases insulin secretion in parallel with an increase in insulin resistance and inflammation [121]. Furthermore, the hippocampal insulin resistance elicited by corticosterone may contribute to the deleterious consequences of hypercortisolism/hyperglycemia observed in T2D [122], i.e. deficits in hippocampal neurogenesis, synaptic plasticity and learning. Such deficits are observed in *d*b/db mouse, a model of T2D [59] in which obesity, hyperglycemia, and elevations in circulating corticosterone levels arise from a mutation that inactivates the leptin receptor [120]. Stranahan et al. [119] studied adrenalectomized db/db mice that were given different doses of corticosterone via drinking water (25 or 250 μg/ml in 0.9% saline). Adrenalectomized *d*b/db mice that had received 25 μg/ml corticosterone replacement learned the location of the hidden platform in the Morris water maze more rapidly than sham–operated db/db mice and adrenalectomized db/db mice receiving 250 μg/ml corticosterone replacement.

In T2D patients, Bruehl et al. [99] measured an increased responsiveness to CRH, coupled with diminished suppression after dexamethasone-responsive test, indicating an abnormality in HPA feedback sensitivity (suspected to be a predictive factor of T2D, [123]), and related to decreased cognitive performance [124], but the brain pathways that mediate these links have not been understood yet. Reynolds et al. [125] showed that morning cortisol levels in elderly people with T2D are high, with deleterious effects on cognitive function. It has moreover been demonstrated that intranasal insulin may normalize stress axis activity in humans by reducing cortisol levels [4]. This inhibitory effect may also contribute to its positive impact on cognitive function. Glycogen synthase kinase 3 β promotes Tau phosphorylation which impairs memory in T2D and is involved in AD, and has been shown to be activated by glucocorticoids [126].

### 3.2 Bioavailability of glucocorticoids: the enzyme 11β-HSD1

The intracellular enzyme 11β-HSD1 catalyzes intracellular regeneration of active glucocorticoids from inert 11-keto forms in liver, adipose tissue and brain [127]. It is widely expressed in hippocampus, cerebellum, and neocortex, suggesting its potential involvement in processes such as memory and learning. Sandeep et al. [128] showed that oral administration of an 11β-HSD1 inhibitor, carbenoxolone, improved verbal fluency in 10 healthy elderly men and improved verbal memory in 12 T2D patients. Quervain et al. showed that a rare haplotype in the 5′ regulatory region of the gene encoding 11β-HSD1 was associated with an increased risk to develop AD [129]. Related to T1D, our group showed elevated level of 11β-HSD1 in the morning urine of T1D children, even in the presence of insulin treatment [130]. This result suggests an alteration of glucocorticoids metabolism associated to T1D.

The important role of hippocampal 11β-HSD1 has recently been demonstrated in a rodent models of aging [131,132]. Indeed, 11β-HSD1 null-mice resist to cognitive decline with aging [127]. Furthermore, treatment of aged rodents with liquorice [133], carbenoxolone, or synthetic selective inhibitors of 11β-HSD1 (for instance UE2316 [131]) improves memory by decreasing local corticosterone concentration in brain [127,134,135] and by preventing brain atrophy [136]. Given our data in T1D children [137], we hypothesized that 11β-HSD1 may also be overactivated in brain of T1D and be responsible for cognitive impairments as it is the case in old individuals. We demonstrated that 11β-HSD1 levels were increased in T1D juvenile rats hippocampus [50,51]. These studies point to a pivotal role for 11β-HSD1 in glucocorticoid excess induced by T1D and consequently of altered hippocampal function (performance on the Y-maze and recognition of a displaced object [51]). An interesting perspective would be to evaluate the potential beneficial effect of 11β-HSD1 inhibition in untreated and insulin-treated diabetic juvenile rats to decipher the role of 11β-HSD1 in hippocampal-dependent cognitive alterations induced by T1D.

### 3.3 Receptors

Brain effects of glucocorticoids are mediated via two types of receptors: the mineralocorticoid receptors (MR) and the glucocorticoid receptors (GR). MR are highly and specifically expressed in hippocampus, basolateral amygdala and prefrontal cortex, a network of brain structures involved in memory and learning processes, while GR are widely expressed throughout the whole brain. Cortisol has a tenfold higher binding affinity for the MR (Kd 0.5 nM) than for the GR (Kd 15–20 nM). Consequently, MR are activated first when cortisol levels increase, followed by GR activation when cortisol levels increase further [138]. In accordance, it has been postulated that cortisol levels follow an inverted U-shaped dose response curve, with very low cortisol levels (predominantly activating MR), as well as very high cortisol levels (activating MR and a large amount of GR) negatively affecting the mediating function of these receptors on information processing (MR/GR balance) [139]. MR activation leads to retrieval of previously learned tasks and behavioral responses to new situations, is neuroprotective, and stimulates hippocampal function [140]. The literature suggests that MR mediate the role of corticosterone in the appraisal of novel situations, behavioral reactivity, and affective responses [100,141], and enhances the performance in spatial hippocampal-dependent cognitive tasks in rodents [142,143] and cognitive performances in human [144]. GR activation is responsible for the consolidation of new information [145], or of a stressful event [146]. A mutation preventing GR from dimerization suggests that gene transcription needs to be activated for the consolidation of memory [147]. Basal corticosterone levels are needed for effective LTP, but higher levels impair it and enhances LTD via GR. High glucocorticoids and stressors also suppress neurogenesis in the dentate gyrus. This U-shaped result may reflect the relative occupancy of MR (at lower doses) and GR (at higher ones) [146,148]. Adrenalectomy also impairs consolidation as does the knockout of GR. However, cognitive performances are improved during chronic blockade of GR with the glucocorticoid antagonist mifepristone (RU486) [149,150].

Only few studies investigated the specific involvement of corticosteroid receptors in altered memory associated to diabetes. For instance, Revsin et al. [150] showed that GR blockade with mifepristone (RU486) improves hippocampal alterations and cognitive impairment in STZ-induced T1D in mice. Interestingly, polymorphisms on the MR and GR-coding genes were recently involved in cognition under stress [151] and AD [152]. Modulating specific activations and inhibitions of MR/GR might be a promising therapeutic way to prevent cognitive impairments induced by diabetes.

## 4 Conclusion

**Fig. 1:**
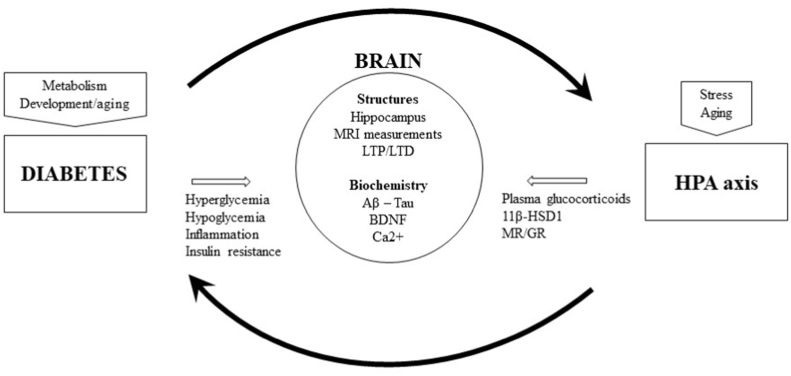
Potential biological targets for the therapeutic approches of the cognitive impairments induced by diabetes. 11β-Hydroxysteroid Deshydrogenase type 1; BDNF: brain derived neurotrophic factor; HPA axis; Hypothalamic pituitary Adrenal axis; LTP/LTD: long-term potentiation/depression; MR/GR mineralocorticoid receptors. MRI: Magnetic Resonance Imaging.

Targeting biological actors involved in HPA axis dysfunctions could be a relevant approach for the treatment of cognitive consequences of diabetes. Each period of life can be concerned. Fig. 1 summarizes the vicious circle that can conduct to cognitive impairments, when brain consequences of diabetes are worsened by glucocorticoid-mediated effects, independently or not from aging or stressful conditions, and from variability of individual resilience (genetic and environmental vulnerability, comorbidities).
